# Time-course analysis reveals that corticosteroids resuscitate diminished CD8+ T cells in COVID-19: a retrospective cohort study

**DOI:** 10.1080/07853890.2020.1851394

**Published:** 2021-01-11

**Authors:** Fangzhou Ye, Jing Liu, Liangkai Chen, Bin Zhu, Li Yu, Boyun Liang, Ling Xu, Sumeng Li, Sihong Lu, Lei Fan, Dongliang Yang, Xin Zheng

**Affiliations:** a Department of Infectious Diseases, Union Hospital, Tongji Medical College, Huazhong University of Science and Technology, Wuhan, China; b Joint International Laboratory of Infection and Immunity, Huazhong University of Science and Technology, Wuhan, China; c School of Public Health, Ministry of Education Key Lab of Environment and Health, Tongji Medical College, Huazhong University of Science and Technology, Wuhan, China; d Intensive Care Unit, The Central Hospital of Wuhan, Tongji Medical College, Huazhong University of Science and Technology, Wuhan, China

**Keywords:** COVID-19, CD8+ T cell, corticosteroids, coagulation, neutrophil to lymphocyte ratio

## Abstract

**Objective:**

To illustrate the effect of corticosteroids and heparin, respectively, on coronavirus disease 2019 (COVID-19) patients’ CD8+ T cells and D-dimer.

**Methods:**

In this retrospective cohort study involving 866 participants diagnosed with COVID-19, patients were grouped by severity. Generalized additive models were established to explore the time-course association of representative parameters of coagulation, inflammation and immunity. Segmented regression was performed to examine the influence of corticosteroids and heparin upon CD8+ T cell and D-dimer, respectively.

**Results:**

There were 541 moderate, 169 severe and 156 critically ill patients involved in the study. Synchronous changes of levels of NLR, D-dimer and CD8+ T cell in critically ill patients were observed. Administration of methylprednisolone before 14 DFS compared with those after 14 DFS (*β* = 0.154%, 95% CI=(0, 0.302), *p*=.048) or a dose lower than 40 mg per day compared with those equals to 40 mg per day (*β* = 0.163%, 95% CI=(0.027, 0.295), *p*=.020) significantly increased the rising rate of CD8+ T cell in 14–56 DFS.

**Conclusions:**

The parameters of coagulation, inflammation and immunity were longitudinally correlated, and an early low-dose corticosteroid treatment accelerated the regaining of CD8+ T cell to help battle against SARS-Cov-2 in critical cases of COVID-19.

## Introduction

The rising pandemic of coronavirus disease 2019 (COVID-19) caused by severe acute respiratory syndrome coronavirus 2 (SARS-CoV-2) has led to worldwide economic losses and mortalities [1]. COVID-19 may lead to deranged coagulation system, dampened immunologic function and inflammatory cytokine release in severe or deceased patients [2,3].

Increasing attention has been focussed on the interplay between inflammation, coagulation and immunity, in which the innate and adaptive immune response might be playing a pivotal role, in the pathogenesis of COVID-19 [4–6]. Severe COVID-19 presented higher D-dimer and was associated with increased probability of developing venous thromboembolism and mortality [7–10]. Based on our previous findings, neutrophil/lymphocyte ratio (NLR), or more specifically, neutrophil-to-CD8(+) T cell ratio (N8R), showed tight correlation with the severity of COVID-19 [3]. Neutrophils are major effectors involved in inflammation, while T lymphocytes were critical cell population in curbing unleashed innate immune response *in vivo* [11]. Therefore, we sought to use NLR, while neutrophil counts often relating to inflammatory activity, and CD8+ T cell as indicators for imbalance between inflammation and antiviral immune response [3,12], at the same time interrogating the representative coagulation parameters, to reveal possible association among coagulation, inflammation and immune system in a longitudinal way by comparing kinetics of these parameters.

Corticosteroids are common and effective medications for suppressing inflammatory activity and attenuate damage from uncontrolled inflammation, which simultaneously induce T lymphocytes apoptosis [13]. Nonetheless, there is no unanimous consensus as to whether corticosteroids should be prescribed to COVID-19 patients [14–17]. Recent evidence suggests that early short course corticosteroids is correlated with reduced rate of respiratory failure, admission to an intensive care unit and mortality [17]. Regarding treatment for thrombosis, heparin is an anticoagulant widely used and is recommended for patients with elevated level of D-dimer [18]. In this study, we aimed to elucidate the effect of these two drugs on the immunologic and coagulation parameters by comparing patients with different initial time and dosage of prescription, which might provide useful information in the therapy for COVID-19.

## Methods

### Study design

This is a retrospective cohort study conducted by two large teaching hospitals in Wuhan, China. The protocol of data collection and laboratory examination were described in our previous study [3]. In brief, patients with COVID-19 admitted to the two hospitals from 1 January 2020 to 16 March 2020 were enrolled in this study. Demographic information, laboratory examination results, day of symptom onset and administrations for every patient were collected from electric health records. The information was stored as records which were defined as any type of data that obtained on the same day for a specific patient. The symptoms were self-reported and were inspected by two experienced clinicians, combined with laboratory tests and radiological findings, to ensure derivations from the COVID-19. We calculated the days from onset of symptoms (DFS) regarding the date of records to align patients in different disease processes for more accurate and sensible analysis. Patients were excluded for unknown date of symptoms onset (Supplementary Figure 1).

### Patient definition and classification

Hypertension was defined according to the JNC report [19]. Cardiovascular disease (CVD) was diagnosed by the guideline of the American College of Cardiology and the American Heart Association [20]. Diabetes mellitus was defined by the criteria proposed by the German Diabetes Association [21]. COVID-19 patients were confirmed by laboratory tests for virus RNA or antibody of SARS-Cov-2, diagnosed and stratified according to the Guidelines of the Diagnosis and Treatment of New Coronavirus Pneumonia (Version 7) released by the National Health Commission of China:

1. *Moderate*: Patients with mild symptoms with or without radiologic findings of pneumonia.
2. *Severe*: Patients who met one of the following criteria (1) short of breath or respiratory rate > 30/min; (2) peripheral oxygen saturation (SpO_2_)≤93%; (3) PaO_2_/FiO_2_≤300 mmHg.
3. *Critically ill*: Patients who met one of the following criteria (1) respiratory failure requiring mechanical ventilation; (2) shock; (3) extra-pulmonary organ failure requiring admission to an intensive care unit.

### Drug dosages

The most commonly used corticosteroid was methylprednisolone (MP) in the treatment for COVID-19 in our cohort. We divided subjects administrated with MP into three groups according to the highest dosages ever prescribed during hospitalizations: low: less than 40 mg/day; medium: 40 mg/day; high: greater than 40 mg/day. Two types of heparins were used: low molecular weight heparin (specifications were 4000, 4100 or 5000 IU) and unfractionated heparin (the specification was 12,500 IU). The duration of therapy was recorded as days of time in total. In the construction of models, the dose of heparin was omitted because most prescriptions used the same doses as the specification of the drug.

### Statistics

Continuous variables were presented as mean (SD) if it is normally distributed, otherwise as median [IQR]. Categorical variables were presented as counts (percentage). Variables among the three groups (moderate, severe and critically ill in ascending order of disease severity) were compared using the Cochran–Armitage trend test and Spearman’s correlation test as appropriate. Skewed variables were logarithmically transformed to obtain better normality in some analyses.

We used generalized additive models to describe the trend of clinical variables and the details of the models were provided in Supplementary materials. Segmented regression was performed by constructing linear mixed models (LMMs) to examine the effects of different administration time of drugs on variation of laboratory parameters. The data were split into two parts according to the time: time ≤14 DFS and time >14 DFS. For models with respect to effect of corticosteroids on CD8+ T cells, we adjusted for the use of thymosin alpha 1 (Tα1), thymopentin (TP5) and intravenous immunoglobulin (IVIG) because of their known effects on T cell development [22–24]. The ages and genders of patients were also deemed influential in the function of the thymus and were included in the models [25,26]. The interaction terms were added to the model to examine the effects of drugs. The construction of LMM was detailed in Supplementary materials. Inverse probability of treatment weights (IPTWs) was developed and used to adjust for the difference between the treatment group (use of MP) and the control group (no use of MP) when exploring the effect of MP on CD8+ T cell population.

A *p* value <.05 was considered to be statistically significant. All analyses were performed in R software (The R Foundation for Statistical Computing, Vienna, Austria).

## Results

A total of 866 patients were involved in the analysis (Supplementary Figure 1). Features of data are summarized in Table 1. Patients aged from 20 to 97 years old and a part of them had comorbid of hypertension and diabetes. Critically ill patients showed higher levels of D-dimer, WBC and NLR.

**Table 1.** Baseline characteristics of enrolled patients.

	Moderate (*n* = 541)	Severe (*n* = 169)	Critically ill (*n* = 156)	*p* Value
*Demographic data*				
Gender female	290 (53.6)	80 (47.3)	68 (43.6)	.017
Age (years)	59.00 [46.00, 67.00]	67.00 [57.00, 73.00]	65.00 [57.00, 75.25]	.926
Death	0 (0.0)	1 (0.6)	30 (19.2)	<.001
DM	61 (12.4)	39 (28.3)	33 (22.3)	<.001
Hypertension	104 (25.0)	52 (48.1)	67 (51.1)	<.001
CVD	36 (7.4)	16 (12.0)	36 (24.5)	<.001
*Laboratory parameters*				
D-dimer (μg/L)	0.41 [0.20, 0.93]	0.96 [0.35, 1.86]	1.04 [0.51, 2.82]	.964
PT (s)	11.70 [11.00, 12.80]	13.25 [12.33, 13.90]	11.90 [11.30, 13.20]	.944
TT (s)	17.40 [16.40, 18.40]	17.60 [16.30, 18.75]	17.70 [17.15, 19.05]	.825
APTT (s)	28.80 [25.80, 34.70]	35.55 [32.02, 38.60]	30.00 [25.75, 35.80]	.921
INR	0.99 [0.95, 1.05]	1.04 [0.98, 1.09]	1.03 [0.97, 1.12]	.961
Fibrinogen (g/L)	2.66 [2.19, 3.59]	4.46 [3.43, 5.35]	3.25 [2.62, 4.10]	.921
WBC (×10^9^/L)	5.45 [4.38, 6.68]	5.67 [4.18, 7.12]	5.86 [4.61, 8.99]	.963
Haemoglobin (g/L)	128.00 [118.00, 139.00]	128.00 [119.00, 139.00]	127.00 [109.00, 137.00]	0.999
Platelet (×10^9^/L)	223.00 [172.00, 291.00]	192.00 [145.00, 274.00]	170.50 [132.25, 262.50]	.933
Neutrophils (×10^9^/L)	3.35 [2.53, 4.47]	3.58 [2.81, 5.44]	4.38 [2.99, 7.40]	.974
Lymphocytes (×10^9^/L)	1.45 [1.06, 1.78]	1.18 [0.80, 1.54]	0.80 [0.53, 1.16]	.963
Monocytes (×10^9^/L)	0.48 [0.37, 0.61]	0.43 [0.33, 0.59]	0.54 [0.38, 0.72]	0.999
NLR	2.27 [1.68, 3.58]	3.15 [2.08, 5.03]	6.07 [3.19, 11.15]	.963
CD3+ T cells (%)	71.81 [65.89, 77.31]	77.86 [77.30, 78.69]	70.30 [61.15, 77.04]	.812
CD4+ T cells (%)	42.11 [36.41, 47.64]	52.59 [47.19, 52.92]	42.27 [37.08, 50.76]	.812
CD8+ T cells (%)	25.00 [20.26, 31.12]	24.80 [23.60, 29.58]	21.78 [15.62, 29.66]	0.999
CD4/CD8 ratio	1.69 [1.20, 2.29]	2.15 [1.68, 2.25]	1.85 [1.23, 2.96]	.812
B cells (%)	12.08 [9.09, 16.25]	4.57 [3.08, 6.06]	10.70 [7.94, 15.14]	.905
NK cells (%)	14.00 [9.08, 20.46]	13.28 [9.50, 17.06]	16.36 [11.54, 21.94]	0.999
CRP (mg/L)	8.85 [3.14, 29.15]	13.80 [3.38, 35.40]	48.40 [38.80, 83.20]	.577
*Medications*				
Tα1/TP5	93 (17.2)	29 (17.2)	44 (28.2)	.006
IVIG	45 (8.3)	19 (11.2)	47 (30.1)	<.001
MP administration	87 (16.1)	49 (29.0)	94 (60.3)	<.001
MP dosage^a^				.677
Medium	73 (83.9)	36 (73.5)	67 (71.3)	
Low	13 (14.9)	9 (18.4)	6 (6.4)	
High	1 (1.1)	4 (8.2)	21 (22.3)	
MP duration	4.00 [2.50, 6.00]	6.00 [5.00, 11.00]	4.00 [2.00, 6.00]	.677
Heparin administration	160 (29.6)	49 (29.0)	117 (75.0)	<.001
Heparin duration	1.00 [1.00, 2.00]	3.00 [1.00, 13.00]	1.00 [1.00, 3.00]	.301

APTT: activated partial thromboplastin time; CVD: cardiovascular disease; CD: cluster of differentiation; CRP: C-reactive protein; DM: diabetes mellitus; INR: international normalized ratio; IVIG: intravenous immunoglobulin; MP: methylprednisolone; NLR: neutrophil to lymphocyte ratio; NK: nature killer; PT: prothrombin time; Tα1/TP5: thymosin alpha 1 or thymopentin; TT: thrombin time; WBC: white blood cell.

Data were counts (percentage) or median [IQR]. *p* Values for Cochran–Armitage trend test or Spearman’s correlation test of the variables among the three groups.

^a^ Highest dosages ever prescribed during hospitalizations: low: less than 40 mg/day; medium: 40 mg/day; high: greater than 40 mg/day.

**Figure 1.**
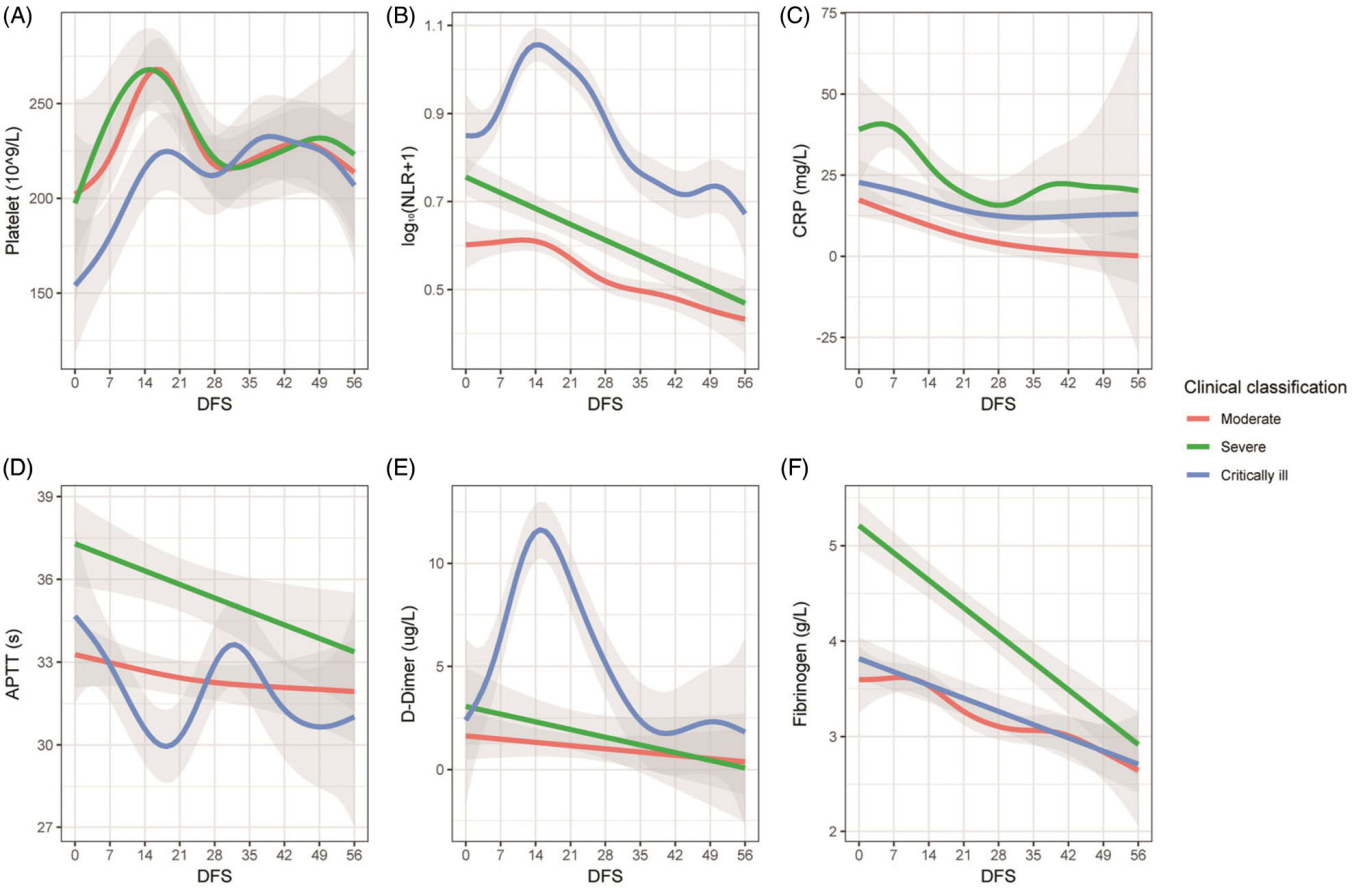
Dynamic changes of coagulation, inflammatory and immunologic indicators in COVID-19 patients. Generalized additive models were used to generate trends of the indicators. Study subjects were stratified as moderate, severe or critically ill. The solid curves or lines represent fitted values of indicators. The upper and lower boundaries of the shaded areas represent 95% CI. APTT: activated partial thromboplastin time; DFS: days from onset of symptoms; NLR: neutrophil/lymphocyte ratio; CRP: C-reactive protein; Log_10_: common logarithm.

**Figure 2.**
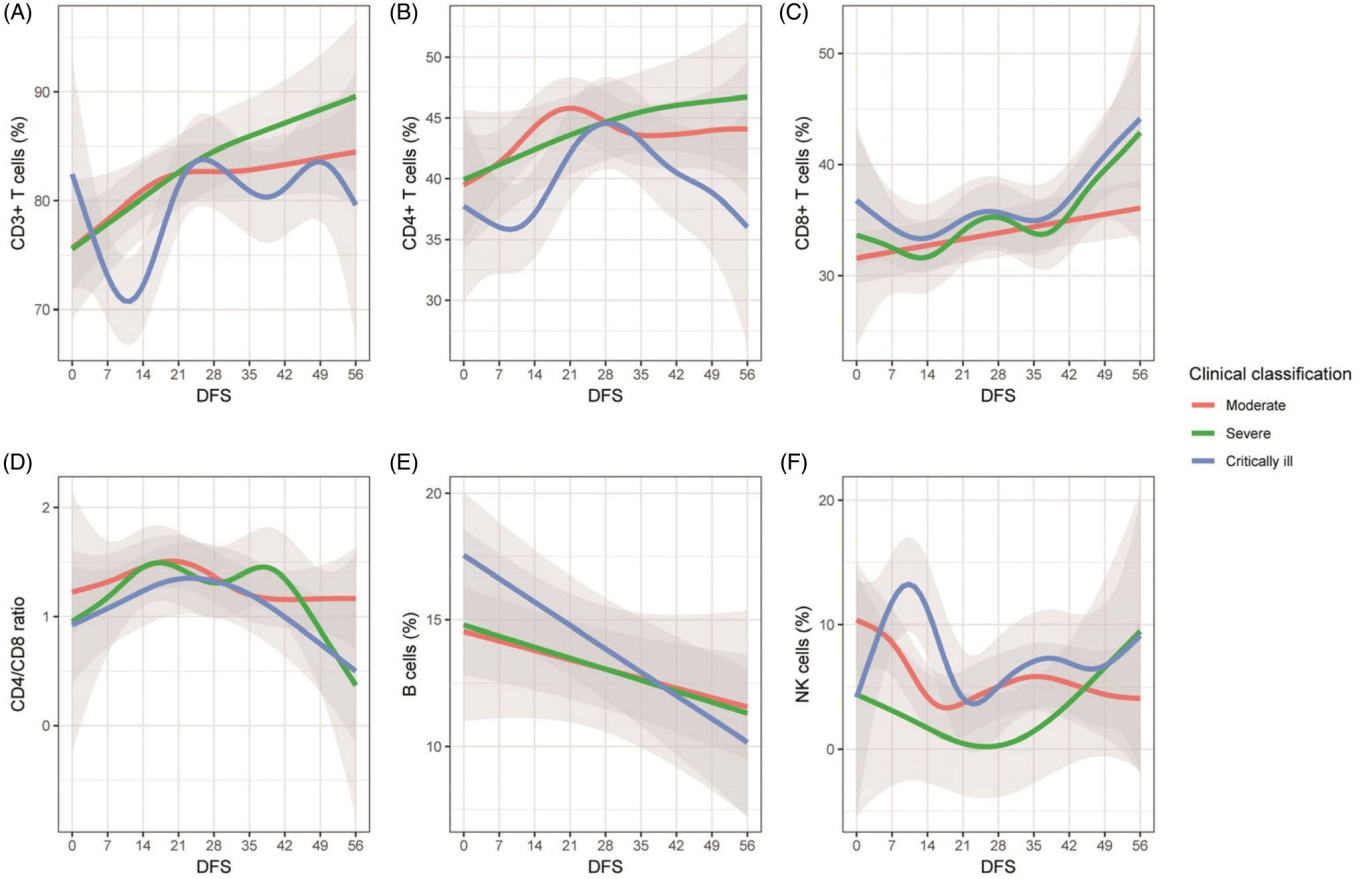
Dynamic changes of T cells and subtypes, B cells and NK cells in COVID-19 patients. Generalized additive models were used to generate trends of the indicators. Study subjects were stratified as moderate, severe or critically ill. The solid curves or lines represent fitted values of indicators. The upper and lower boundaries of the shaded areas represent 95% CI. DFS: days from onset of symptoms.

As can be seen, concomitant changes of NLR, activated partial thromboplastin time (APTT) and D-dimer were observed in critically ill patients between 0 and 14 DFS (Figure 1(B–E)). Both NLR and D-dimer increased on the first day of onset of symptoms, peaking at 14 DFS and declined between 14 and 35 DFS. It is noteworthy that level of APTT in the same patient group decreased in the first two weeks and turned to rising trend in the 14–35 DFS time frame. The levels of C-reactive protein (CRP) elevated in the first week, peaking at seven DFS and then declined in 7–28 DFS in critically ill patients. The platelet counts increased in the first two weeks, peaked at 14 DFS, and declined between 14 and 28 DFS for all three groups of patients (Figure 1(A)). Based on the findings of NLR, we further analysed the kinetic changes of different lymphocyte subsets and NK cells, results showed that CD8+ T cells in critically ill patients varied synchronously compared with NLR, D-dimer and APTT in 0–28 DFS, as they shared similar inflection points around 14 DFS (Figures 1(B,E) and 2(C)).

**Figure 3.**
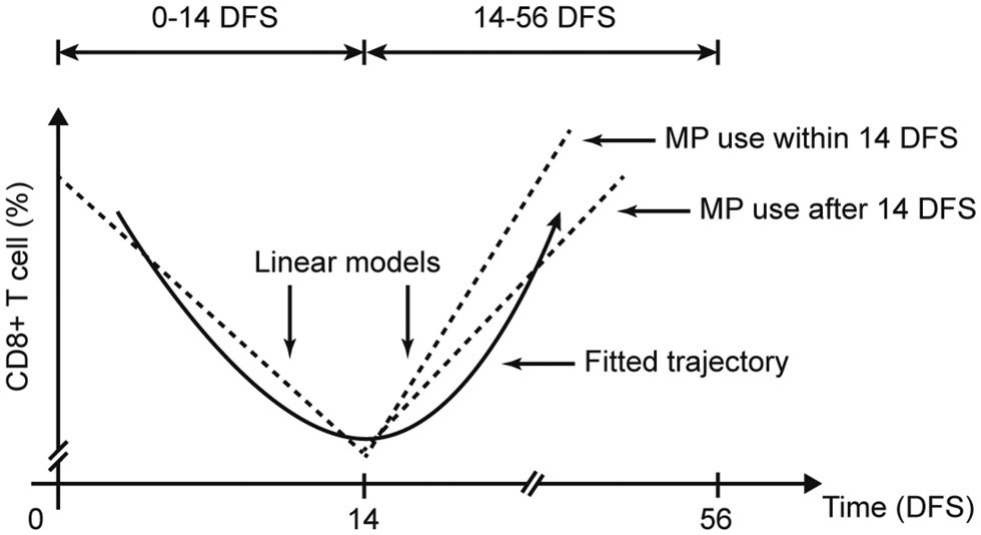
A schematic diagram of different administration time of methylprednisolone (MP) affecting the time course of CD8+ T cell. The *x*-axis is days from onset of symptoms (DFS) and *y*-axis the percentage of CD8 T cell. The solid curve indicates the trajectory of CD8 T cell percentage along the timeline. The dashed lines on the left and right part separated by 14 DFS indicate the linear mixed models fitted by data from 0 to 14 DFS and 14 to 56 DFS. Administration of MP within 14 DFS significantly increased the growth rate of CD8+ T cell percentage of patients after 14 DFS, compared with those who received MP therapy after 14 DFS.

In order to explore the impact of glucocorticoids (GCs) on CD8+ T cells, two classes of separated linear models were constructed, one for the descending part and the other one for the ascending part of the trajectory of CD8+ T cell, as divided by 14 DFS (Figures 2 and 3). We first explored the effect of MP on CD8+ T cell between the treated and the untreated and found that MP did not significantly influence the trend of CD8+ T cell both in 0–14 DFS and 14–56 DFS (Supplementary Table 1). We further examined the interactive effect between administration of MP within 14 DFS and the time. We first established a model involved only time which showed significant correlation with response in both 0–14 DFS (*β*=–0.200, *p*=.052) and 14–56 DFS (*β* = 0.101, *p*<.001) (Table 2). The univariate model was further adjusted for patients’ age, gender, complications, dosage and duration of therapy, and use of other drugs exerting potential effect on CD8+ T cell. An MP administration started within 14 DFS significantly boosted the recovery of CD8+ T cell in 14–56 DFS time frame with an increment of growth rate of 0.154% per day (*p*=.048), compared with patients administrated after 14 DFS. However, the decrease of CD8+ T cells in 0–14 DFS was not significantly accelerated by an MP administration started within 14 DFS in both models (*β*= −0.525, *p*=.708). Taken together, the results suggested that MP administration in 0–14 DFS do not significantly affect the decreasing rate of CD8+ T cell in 0–14 DFS but aid in the recovery of it in 14–56 DFS, compared with those administrated in 14–56 DFS. A significant increment of the growth rate of CD8+ T cell in 14–56 DFS was also observed in patients administrated with low dose of MP compared with those in a medium dose group (*β* = 0.163, *p*=.020).

**Table 2.** The linear mixed model of CD8+ T cells percentage in response to the time and administration start time of methylprednisolone.

	0–14 DFS			14–56 DFS		
	*β*	95% CI	*p* Value	*β*	95% CI	*p* Value
*Unadjusted model*						
Time	–0.035	(–2.041, 1.979)	.973	0.003	(–0.098, 0.105)	.955
MP	4.019	(–20.341, 28.423)	.750	–0.190	(–5.136, 4.764)	.940
Time:MP	–0.165	(–2.23, 1.893)	.876	0.116	(–0.027, 0.257)	.113
*Adjusted model*						
Time	0.113	(–2.336, 2.841)	.934	–0.002	(–0.146, 0.142)	.978
MP	10.861	(–19.530, 44.416)	.520	–1.661	(–6.622, 3.422)	.525
Severe^a^	5.913	(–1.532, 13.374)	.157	2.006	(–2.069, 6.081)	.351
Critically ill^a^	4.094	(–1.501, 9.688)	.191	4.264	(0.570, 7.960)	.030
Age	2.663	(–2.280, 7.601)	.335	–0.252	(–3.636, 3.134)	.888
Gender female	1.177	(–7.965, 10.33)	.817	–2.818	(–7.945, 2.316)	.298
Had hypertension	–0.314	(–0.472, −0.157)	.001	–0.270	(–0.388, −0.153)	<.001
Had CVD	4.224	(–0.212, 8.656)	.091	0.804	(–2.187, 3.796)	.610
Time:MP	–0.525	(–3.322, 2.002)	.708	0.154	(0, 0.302)	.048
Time:Tα1/TP5	0.034	(–0.495, 0.564)	.909	0.047	(–0.05, 0.146)	.357
Time:IVIG	0.246	(–0.257, 0.749)	.381	–0.048	(–0.143, 0.049)	.346
Time:low dosage^b,c^	0.606	(–0.072, 1.286)	.112	0.163	(0.027, 0.295)	.020
Time:high dosage^b,c^	–0.422	(–1.050, 0.206)	.230	0.036	(–0.14, 0.21)	.697
Time:duration of therapy^c^	0.012	(–0.037, 0.061)	.661	–0.004	(–0.012, 0.004)	.340

*β*: estimated coefficient; CI: confidence interval; CVD: cardiovascular disease; MP: methylprednisolone; Tα1/TP5: thymosin alpha 1 or thymopentin; IVIG: intravenous immunoglobulin.

Colons (:) denote interaction of the two variables. The MP terms in the model represent a medication of MP in patients started within 14 days from the onset of symptoms (DFS), with those started after 14 DFS as a reference category. The Tα1/TP5 term denotes either use of thymosin alpha 1 or thymopentin. The IVIG term denotes the use of intravenous immunoglobulin. The unit of time was DFS.

^a^ Stratifications of patients with moderate type considered as a reference category.

^b^ Dosages of drugs with medium dosage considered as a reference category.

^c^ These variables refer to the use of MP.

Similar analysis was also conducted with respect to heparin and the dynamic change of D-dimer. No significant effect was observed in 0–14 and 14–56 DFS time frames for a patient administrated heparin within 14 DFS on D-dimer (Table 3).

**Table 3.** The linear mixed model of levels of D-dimer in response to the time and administration start time of heparin.

	0*–*14 DFS			14–56 DFS		
	*β*	95% CI	*p* Value	*β*	95% CI	*p* Value
*Unadjusted model*						
Time	0.334	(–0.147, 0.82)	.181	–0.172	(–0.238, −0.105)	<.001
Heparin	2.087	(–4.991, 9.134)	.567	1.543	(–3.639, 6.759)	.562
Time:heparin	–0.139	(–0.813, 0.545)	.691	–0.135	(–0.363, 0.09)	.244
*Adjusted model*						
Time	0.366	(–0.279, 1.037)	.294	–0.171	(–0.265, −0.081)	.001
Heparin	2.416	(–5.798, 11.639)	.602	0.577	(–4.995, 6.573)	.848
Severe^a^	1.805	(–3.188, 6.760)	.524	0.541	(–2.000, 3.191)	.708
Critically ill^a^	7.726	(2.772, 12.430)	.007	5.958	(3.143, 8.636)	<.001
Age	0.055	(–0.079, 0.175)	.442	0.069	(–0.003, 0.140)	.091
Gender female	–2.759	(–6.801, 1.298)	.234	–1.380	(–3.585, 0.805)	.263
Had hypertension	–0.221	(–3.977, 3.869)	.920	1.989	(–0.408, 4.337)	.137
Had CVD	–5.742	(–9.826, −1.466)	.021	–4.670	(–7.818, −1.407)	.012
Time:heparin	–0.147	(–1.043, 0.675)	.745	–0.108	(–0.376, 0.135)	.410
Time:duration of therapy^b^	0.013	(–0.004, 0.032)	.206	–0.001	(–0.005, 0.003)	.527

*β*: estimated coefficient; CI: confidence interval; CVD: cardiovascular disease.

Colons (:) denote interaction of the two variables. The heparin terms in the model represent a medication started within 14 days from the onset of symptoms (DFS), with those started after 14 DFS as a reference category. The unit of time was DFS.

^a^ Stratifications of patients compared with moderate type.

^b^ This variable refers to the use of heparin.

## Discussion

In this longitudinal cohort study, we have found that COVID-19 presented trends towards transient hypercoagulability in critically ill patients, while time-course correlation of levels of NLR, D-dimer, APTT and CD8+ T cell was observed. NLR and D-dimer increased in the first two weeks since onset of symptoms, accompanied by the decline of CD8+ T cells. Early low-dose GC use, specifically, a dose lower than 40 mg within 14 days since onset of symptoms, might benefit the recovery of CD8+ T cell in the convalescent phase of COVID-19 patients.

Although it is unclear for the order as to which of the NLR and D-dimer elevated first, it is possible that disequilibrium of immune response evidenced by NLR, be it innate or adaptive, was highly correlated with the timing of thrombosis, combined with a hypercoagulable state and inflammatory activity in COVID-19 patients. A similar but negatively varying pattern in CD8+ T cell, a dominant subtype of T cells declined in lymphocytopenia, was also recorded [3]. Although conclusive evidence is needed to clearly demonstrate the underlying mechanisms, it is plausible to speculate an association between the occurrence of abnormal coagulation and immune system, mediated by extensive inflammation in these individuals. Inflammatory injury would activate coagulation cascade and immune response. Also, emphasis should be placed on the CD8+ T cell, which was the most prominent functional cell with decreased magnitude in the course of immune response against the infection. Future study is required to address the issue of whether SRAS-Cov-2 infection would lead to coagulation abnormality via host immunity.

We sought to explain the time-varying patterns by conditioning on the relevant factors, including selecting separated time frames for a linear approximation, to attenuate bias of confounders. In this cohort with relatively larger population, the prime trend of CD8+ T cells percentage in critically ill patients was similar to our previous findings [3]. Since T lymphocytes constitute adaptive immunity against viral infection and its development is subjected to administration of GC, an anti-inflammatory medication remained controversial in the treatment of COVID-19 [13,15,16,27]. We examined the effect of administration time of GC on CD8+ T cells change and discovered that patients received early use of GC showed significantly faster rate of regaining of transiently declined CD8+ T cell in circulation, at the same time without significantly affecting the decreasing rate in the early phase of disease. The result suggested that early GC therapy might benefit patients with signs of transforming into worse condition without inducing suppression on CD8+ T cell, which is a major component in adaptive immunity fighting against viral infection. It is noteworthy that low dose of GC, less than 40 mg per day in this study, should strike a balance between anti-inflammation and the immunosuppression. The benefits of early low-dose GC resembled the results obtained by Fadel et al. (0.5 to 1 mg/kg/day, duration varied from 3 to 7 days) [17]. Instead, we have shown that dynamic change of number of CD8+ T cell is subjected to the timing of GC administration, suggesting early GC use might benefit the patients in a way with respect to immune function reflected by the quantity of its effectors. In addition, start time of administration of heparin posed no significant influence on the trend of D-dimer, indicating that heparin should be used according to specific clinical settings and not determined by an exact time frame.

Selection bias, with sicker patients more likely to receive more potent therapies, is a common reason for affecting the conclusions of drug effects study. Therefore, we included stratifications of patients and the use of potentially confounding drugs, herein the thymosin alpha 1, thymopentin and IVIG, as a covariate in the models, and incorporated IPTWs to attenuate the deviations.

Limitations exist in our study. First, this study is a retrospective analysis and has inherent limitations of a retrospective design and prospective interventional trials are needed to verify the relationship between GC and CD8+ T cell. Second, due to the limited experimental conditions, the characteristics of the lymphocytic cell population are not examined in depth. Third, because some response variables cannot be matched up with available distribution functions, several indeterminate GAMs were obtained in our analyses and were not able to be properly interpreted. Last but not least, we did not include inflammatory cytokines in our analysis because adequate analyses have been performed for cytokines in our previous research.

In conclusion, we have displayed time-course correlations of clinical indicators of inflammation, coagulation and immunologic parameters in a cohort of in-patients with COVID-19, which might be helpful in revealing synergistic relationship among them. Our data suggested use of GC as and when appropriate is beneficial to the regaining of CD8+ T cell in later phase of COVID-19, compared with those who were administrated 14 days later from onset of symptoms. Timely anti-inflammatory medications are of importance if necessary. Nonetheless, further study addressing the issue of appropriate time and duration for use of anti-coagulation therapy is warranted.

## Supplementary Material

Supplemental Material
