# Supplementary material for: Time-course analysis reveals that corticosteroids resuscitate diminished CD8+ T cells in COVID-19: a retrospective cohort study
Source: Ann Med. 2021 Jan 11;53(1):181–8. doi: 10.1080/07853890.2020.1851394 (PMC7877944; doi:10.1080/07853890.2020.1851394)
Supplement: Supplemental Material [file IANN_A_1851394_SM0325.docx]

**Supplementary materials**

**Time-course analysis reveals that corticosteroids resuscitate diminished CD8+ T cells in COVID-19**

Fangzhou Ye, Jing Liu, Liangkai Chen, Bin Zhu, Li Yu, Boyun Liang, Ling Xu, Sumeng Li, Sihong Lu, Lei Fan, Dongliang Yang, Xin Zheng

**Table of contents**

Supplementary methods

Supplementary references

Supplementary Table 1

Supplementary Figure 1

**Supplementary methods (R software function names in italic)**

**Generalized additive models**

Time course of variables were displayed according to the days from onset of symptoms (DFS). We excluded patients with dates of records later than 45 DFS to reduce confounding from unreliable data **(Supplementary Figure 5)**. Generalized additive models (GAM) were established to show the trends of the investigated variables, which were adjusted for confounders (Age, gender, complications and medication) and variations at personal level. The curves could be considered as the averages of the variables at certain points on the timeline (1, 2). The distribution of dependent variable was assumed to be Gaussian distribution and linked by identity function. The inter-person effect was addressed by specifying a random term by a unique serial number of patient in the model. For trends of laboratory parameters, replicated models were fitted for each group of patients. Grouping factors were added as a main effect to avoid missing the intercepts from patients with different clinical classifications, because the smooth functions were zero-centered. Thin plate regression spline was used to fit non-linear relationship between the time and dependent variable. The dimension of the spline function was set to the default value of 10, because most resulted functions were found to have an estimated degree of freedom (edf) less than 10. The formula used in GAM was listed below:

$$Y_{t}=CC+Age+Gender+Complications+Medications+s\left(DFS\right)+\varepsilon_{i}$$

In the formula, $Y_{t}$ denotes the expectation of response variable;$CC$ denotes the clinical classification; $Complications$ includes presence of diabetes, hypertension and cardiovascular disease; $Medications$includes use of methylprednisolone and heparin; $s\left(DFS\right)$ denotes the smooth term of time, i.e. days from onset of symptoms;$\varepsilon_{i}$is the random effect between subjects.

Fitted values were extracted by *predict_gam* excluding all parametric covariates by R package tidymv. The upper and lower boundaries of curves, delineated as shaded areas, were calculated as fitted values plus or minus 1.96 times standard error. Narrower shaded area indicated higher confidence of the fitted values. Smooth terms with p>0.05 were not statistically significant and therefore the corresponding curves provide information of nothing but random perturbation of the response variable along the timeline.

**Linear mixed models**

To examine whether drug prescription time affects trajectory of variables, linear mixed model was constructed by R package *lme4* (3). The models were fitted by restricted maximum likelihood and the t test was performed by Satterthwaite's method with *lmerTest* (4)*.* We also included random effects at personal level to account for variability from different patients, assuming that the difference only affects the intercept (i.e. the average of the variable at the first day when symptom was observed).

**Supplementary references**

1. Wood SN. Generalized Additive Models An Introduction with R SECOND EDITION ISSN: 978-1-4987-2833-1.

2. Wood SN. Fast stable restricted maximum likelihood and marginal likelihood estimation of semiparametric generalized linear models. *Journal of the Royal Statistical Society Series B-Statistical Methodology.* 2011;73:3-36.

3. Bates D, Maechler M, Bolker BM, and Walker SC. Fitting Linear Mixed-Effects Models Using lme4. *Journal of Statistical Software.* 2015;67(1):1-48.

4. Kuznetsova A, Brockhoff PB, and Christensen RHB. lmerTest Package: Tests in Linear Mixed Effects Models. *Journal of Statistical Software.* 2017;82(13):1-26.

| **Supplementary Table 1. The linear mixed model of CD8+ T cells percentage in response to the time and administration of methylprednisolone.** | | | | | | | |
| --- | --- | --- | --- | --- | --- | --- | --- |
|  | 0-14 DFS | | |  | 14-56 DFS | | |
|  | β | 95% CI | p-value |  | β | 95% CI | p-value |
| **Unadjusted Model** |  |  |  |  |  |  |  |
| Time | -0.196 | (-0.451, 0.058) | 0.135 |  | 0.125 | (0.083, 0.167) | <0.001 |
| MP | 0.635 | (-4.089, 5.361) | 0.793 |  | 2.112 | (-0.508, 4.731) | 0.115 |
| Time : MP | 0.010 | (-0.419, 0.438) | 0.964 |  | -0.071 | (-0.146, 0.004) | 0.064 |
| **Adjusted Model** |  |  |  |  |  |  |  |
| Time | -0.145 | (-0.145, -0.133) | 0.462 |  | 0.114 | (0.114, 0.115) | <0.001 |
| MP | 2.531 | (2.526, 2.740) | 0.437 |  | 0.668 | (0.667, 0.703) | 0.650 |
| Time : MP | -0.181 | (-0.181, -0.162) | 0.563 |  | -0.023 | (-0.023, -0.022) | 0.584 |
| Colons (:) denote interaction of the two variables. The MP terms in the model represent the use of MP. The Tα1/TP5 term denotes either use of thymosin alpha 1 or thymopentin. The IVIG term denotes the use of intravenous immunoglobulin. The unit of time was DFS. The model was adjusted for age, gender, clinical classification, presence of hypertension or CVD, use of thymosin alpha 1 or thymopentin, and use of IVIG. ^a^: Stratifications of patients with moderate type considered as a reference category.  ^b^: Dosages of drugs with medium dosage considered as a reference category.  ^c^: These variables refer to the use of MP.  β = estimated coefficient, CI = confidence interval, CVD = cardiovascular disease, MP = methylprednisolone, Tα1/TP5 = thymosin alpha 1 or thymopentin, IVIG = intravenous immunoglobulin. | | | | | | | |


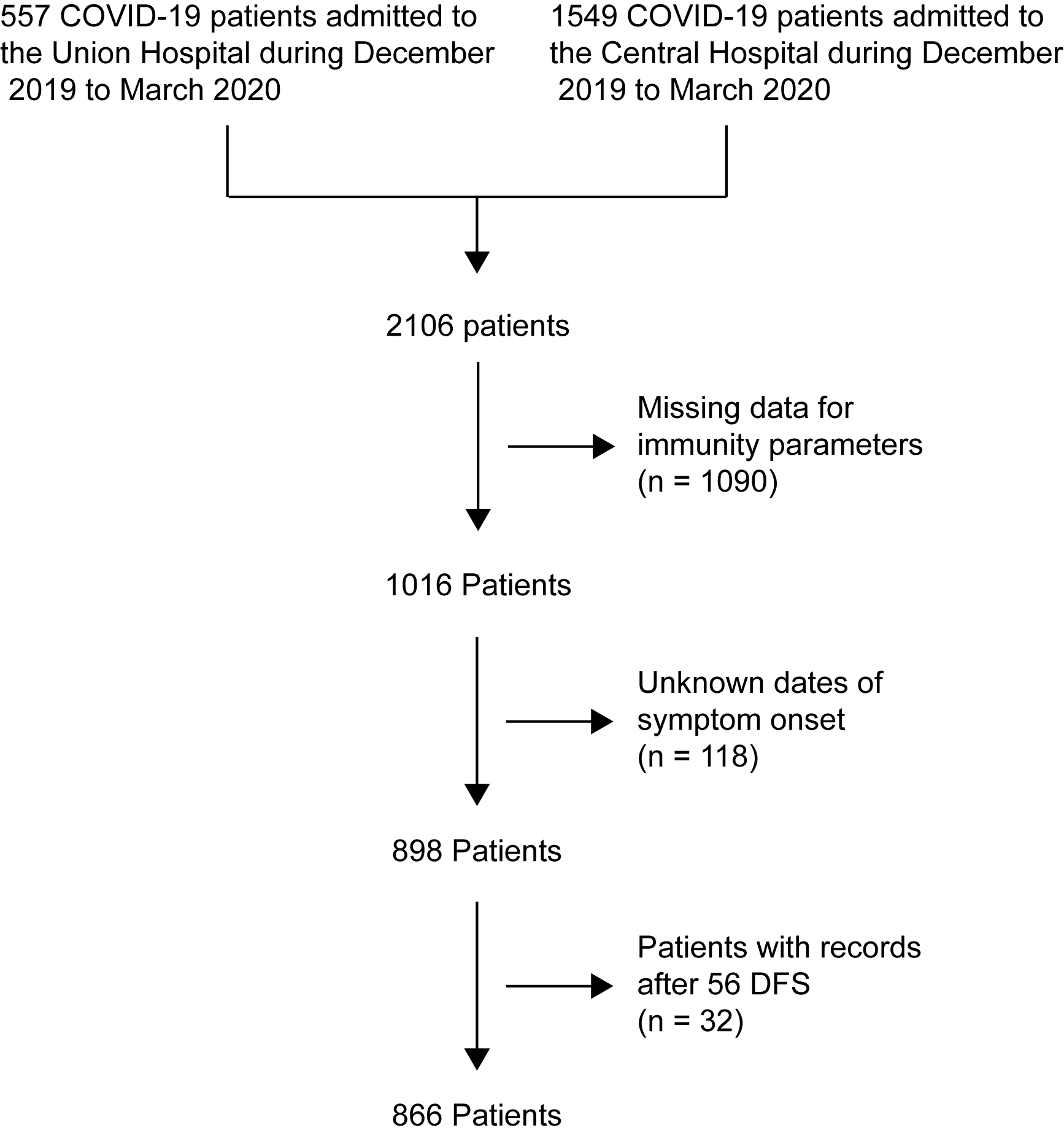


**Supplementary Figure 1. The flow diagram showing enrollment of patients in this study**

Data in the parentheses were number of patients (n) and number of records (N). DFS = days from onset of symptoms.
